# Design of thienopyranone-based BET inhibitors that bind multiple synthetic lethality targets

**DOI:** 10.1038/s41598-020-68964-6

**Published:** 2020-07-21

**Authors:** Kendra R. Vann, Dhananjaya Pal, Guillermo A. Morales, Adam M. Burgoyne, Donald L. Durden, Tatiana G. Kutateladze

**Affiliations:** 1 Department of Pharmacology, University of Colorado School of Medicine, Aurora, CO USA; 2 Division of Pediatric Hematology and Oncology, Department of Pediatrics, Moores Cancer Center, University of California San Diego, La Jolla, CA USA; 3 SignalRx Pharmaceuticals, San Diego, CA USA; 4 Division of Hematology-Oncology, Department of Medicine, Moores Cancer Center, University of California San Diego, La Jolla, CA USA

**Keywords:** NMR spectroscopy, X-ray crystallography, Drug development

## Abstract

Development of small molecule compounds that target several cancer drivers has shown great therapeutic potential. Here, we developed a new generation of highly potent thienopyranone (TP)-based inhibitors for the BET bromodomains (BDs) of the transcriptional regulator BRD4 that have the ability to simultaneously bind to phosphatidylinositol-3 kinase (PI3K) and/or cyclin-dependent kinases 4/6 (CDK4/6). Analysis of the crystal structures of the complexes, NMR titration experiments and IC_50_ measurements reveal the molecular basis underlying the inhibitory effects and selectivity of these compounds toward BDs of BRD4. The inhibitors show robust cytotoxic effects in multiple cancer cell lines and induce cell-cycle arrest and apoptosis. We further demonstrate that concurrent disruption of the acetyllysine binding function of BRD4 and the kinase activities of PI3K and CDK4/6 by the TP inhibitor improves efficacy in several cancer models. Together, these findings provide further compelling evidence that these multi-action inhibitors are efficacious and more potent than single inhibitory chemotypes.

## Introduction

The bromodomain and extra-terminal domain (BET) proteins have emerged as promising pharmacological targets for the treatment of a wide array of human diseases, particularly inflammation and cancer^1–5^. Of the four members of the BET family proteins, BRD4 is the most thoroughly characterized, and its aberrant activity has been associated with NUT midline carcinoma, colon, breast and prostate cancers, neuroblastoma, and hematopoietic cancers. BRD4 overexpression has been shown to promote tumor growth by stimulating transcription of major oncogenic drivers, such as MYC. Most recently BRD4 has been identified as the human protein to which the protein E from the SARS-CoV-2 virus binds^6^. BRD4 contains two bromodomains, BRD4_BD1_ and BRD4_BD2_, that recognize acetylated lysine residues in histones and non-histone proteins. Specifically, BRD4_BD1_ binds to acetylated histones, including poly-acetylated histone H4, recruiting and/or stabilizing BRD4-containing transcription complexes at target gene promoters and enhancers^7^, whereas the second bromodomain, BRD4_BD2_, is also capable of associating with acetylated non-histone proteins^8^.

A wide array of inhibitors of BRD4 as well as pan-BET inhibitors have been developed and are currently being tested in clinical trials, yet there is a pressing need to improve the efficacy of inhibition. BET inhibitors are known to produce variable anti-proliferative effects, and cancer cells often develop resistance to the drugs through activating compensating survival signaling pathways that bypass the drug action^9–12^. These limitations can be overcome through concurrent targeting multiple signaling pathways that support cancer growth and characterized by synthetic lethality relationship. This could be achieved through applying a combination of synergistic individual inhibitors or using a single molecule capable of binding to multiple targets. A combined therapy through inhibiting BRD4 and PI3K by a single molecule has been shown to produce potent antitumor effects in several cancer models driven by MYC^13–15^. In addition to reducing tumor growth, inhibition of both BRD4 and PI3K reduces the infiltration of myeloid-derived suppressor cells and stimulates antitumor immune responses^16^. Dual and triple activity inhibitors specific for BET and other known oncogenic drivers, including cyclin-dependent kinases (CDK), MAPK, PLK1, HDAC, CBP/p300, and ALK are being developed^17–21^.

In this study, we report the biological activities and mechanisms of action of thienopyranone (TP)-based inhibitors with increased potency toward BRD4 and selectivity toward BRD4_BD1_. The cancer models data demonstrate that simultaneous disruption of the acetyllysine binding function of BRD4 and kinase activities of PI3K and CDK4/6 improves efficacy. Our findings provide molecular and structural bases to advance TP-based compounds toward the development of novel anti-cancer therapies and critical tools to characterize the role of BRD4 in viral infections, including SARS-CoV types.

### Results and discussion

#### Design of TP inhibitors

**Figure 1:**
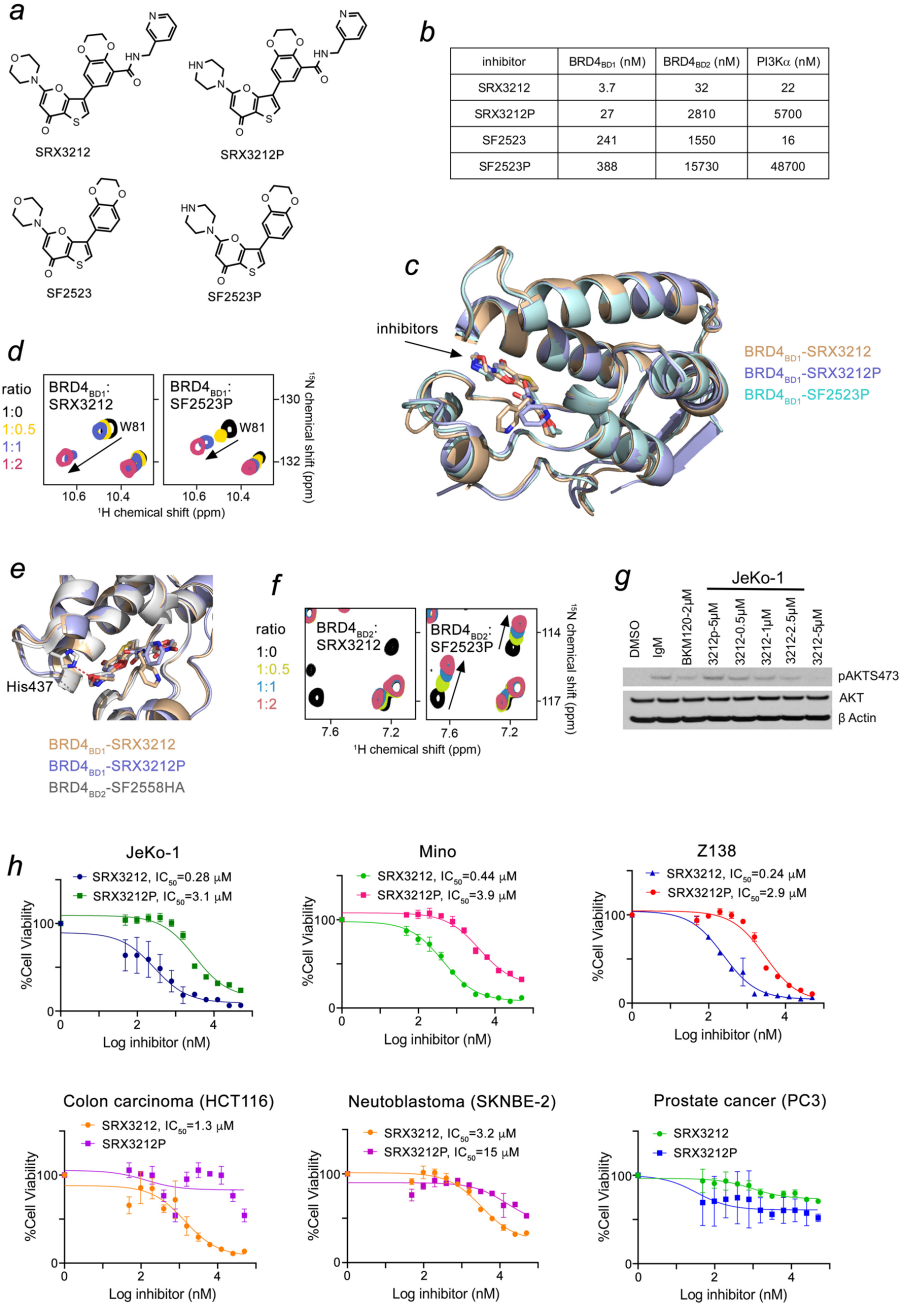
Molecular basis for the enhanced potency and selectivity of the TP inhibitors. (**a**) Chemical structures of the inhibitors. (**b**) IC_50_ values, measured by displacement binding assays for BRD4 BDs or a kinase screening assay for PI3K. Values for SF2523 are taken from^13^. (**c**) Overlay of crystal structures of BRD4_BD1_ in complex with SRX3212 (tan), SRX3212P (purple) and SF2523P (cyan). (**d**) Superimposed ^1^H,^15^N HSQC spectra of uniformly ^15^N-labeled BRD4_BD1_, recorded while the indicated inhibitors were titrated in. The spectra are color-coded according to the protein:inhibitor molar ratio. (**e**) Overlay of crystal structures of BRD4_BD1_ in complex with SRX3212 (tan) and SRX3212P (purple) and of BRD4_BD2_ in complex with SF255HA (gray) (PDB ID: 5U2C). (**f**) Superimposed ^1^H,^15^N HSQC spectra of uniformly ^15^N-labeled BRD4_BD2_, recorded while the indicated inhibitors were titrated in. The spectra are color-coded according to the protein:inhibitor molar ratio. (**g**) Western blot analysis of AKT phosphorylation at Ser473 after treatment of JeKo-1 cells with SRX3212, SRX3212P or BKM120 (pan PI3K inhibitor). (**h**) Anti-proliferative activity of SRX3212 and SRX3212P against indicated cancer cell lines assessed by CellTiter-Glo assays.

We have previously described thienopyranone-based inhibitors, including SF2523 that binds both BRD4 and PI3K and inhibits tumor growth and metastasis^13^. In effort to enhance its potency, we designed a new set of chemotypes by in silico screening. Docking the modified thienopyranone scaffold to BRD4_BD1_ and BRD4_BD2_ suggested that substituents in the benzodioxane moiety of SF2523, such as the pyrid-3-ylmethylaminocarbonyl group, might increase binding to BRD4 BDs, whereas replacing the morpholino ring in SF2523 with the piperazine ring might provide selectivity for BRD4_BD1_ (Fig. 1a). These compounds were synthesized and their activities toward BRD4_BD1_ and BRD4_BD2_ were assessed in a displacement binding assay using the polyacetylated histone H4 peptide H4K5acK8acK12acK16ac as a ligand (Fig. 1b). Comparison of IC_50_ values of SRX3212 and the parent compound SF2523 revealed a notable difference in potency for BRD4. SRX3212 displayed an IC_50_ of 3.7 nM toward BRD4_BD1_ and 32 nM toward BRD4_BD2_, and therefore was ~ 65-fold more potent inhibitor of BRD4_BD1_ and ~ 48-fold more potent inhibitor of BRD4_BD2_ than SF2523. Importantly, SRX3212 retained its high inhibitory activity for PI3Kα as measured by a kinase screening assay (Fig. 1b). These data demonstrate that the pyrid-3-ylmethylaminocarbonyl benzodioxane moiety markedly increases potency of the TP-based compounds for BRD4 without compromising their activity toward PI3K.

Consistent with the docking screening, replacement of the morpholino moiety in SF2523 with the piperazine moiety in SF2523P led to a substantial, ~ 40-fold increase in selectivity toward BRD4_BD1_ over BRD4_BD2_ compared to a ~ sixfold selectivity of the parent compound SF2523. The IC_50_ measurements showed that adding the piperazine substituent does not significantly alter inhibition of BRD4_BD1_ but substantially decreases inhibition of BRD4_BD2_. Further modification of SRX3212 into SRX3212P yielded the most selective inhibitor capable of distinguishing BRD4_BD1_ over BRD4_BD2_ by two orders of magnitude. The ability of piperazine-containing compounds SRX3212P and SF2523P to associate and inhibit PI3Kα was also decreased as measured by kinase screening assays.

#### Structural basis for high potency toward BRD4_BD1_

To gain insight into the molecular mechanism underlying high potency of the inhibitors toward BRD4_BD1_, we co-crystallized BRD4_BD1_ with SRX3212, SRX3212P and SF2523P and determined the crystal structures of the complexes to 1.8–2.7 Å resolution (Fig. 1, Suppl. Figure 1, and Suppl. Table 1). An overlay of the structures showed that all inhibitors occupy the same deep hydrophobic pocket located at the top of the four-helix bundle of BRD4_BD1_ (Fig. 1c and Suppl. Figure 2). The BRD4_BD1_ domain folds superimpose very well, and only small differences were observed in the position of the ZA and BC loops and of the water shell molecules, lining the binding pockets. Analysis of the complexes within 5 Å of the bound compounds revealed several conserved features. In all complexes, the thienopyranone moiety inserts deeply in the binding pocket laying parallel to the α-helices of BRD4_BD1_, whereas the benzodioxane moiety of SRX3212, SRX3212P and SF2523P is oriented toward the ZA loop of BRD4_BD1_. The hydrophobic interactions of the thienopyranone scaffold with Val87, Leu92, Leu94 and Ile146 and the Trp81-Pro82-Phe83 helical turn in the ZA loop stabilize the complexes (Suppl. Figure 1). The carbonyl oxygen in the thienopyranone moiety of all compounds forms a hydrogen bond with the amide nitrogen of Asn140 and a water-mediated hydrogen bond with the hydroxyl group of Tyr97 of BRD4_BD1_. The hydrogen bonds involving Asn140 and Tyr97 represent the hallmark contacts of bromodomains with physiological ligands, the acetyllysine containing sequences, such as H4K5acK8ac, and therefore the thienopyranone chemotypes act as acetyllysine mimetics.

**Figure 2:**
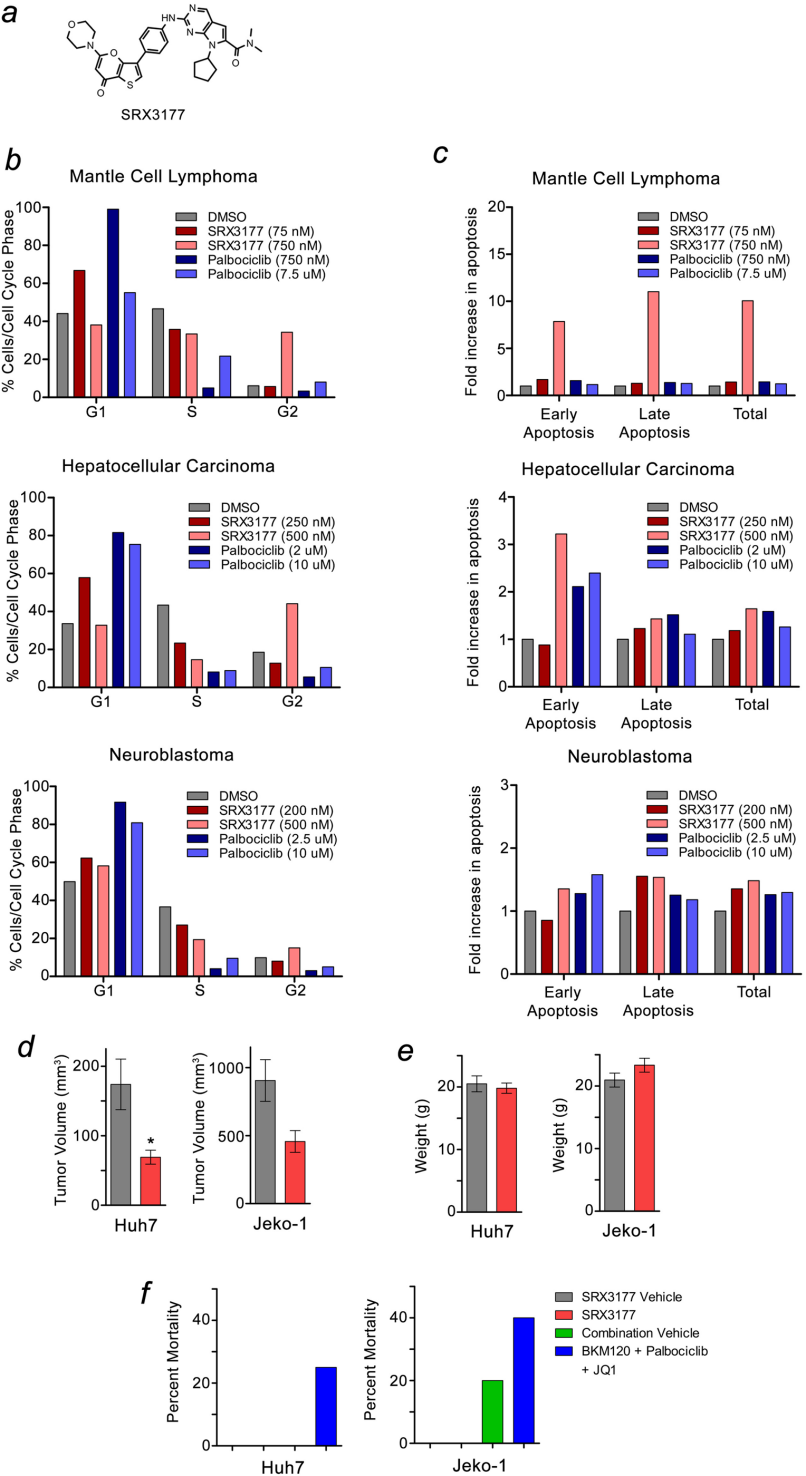
TP inhibitor SRX3177 induces apoptosis and cell cycle arrest and has in vitro efficacy in mantle cell lymphoma, hepatocellular carcinoma and neuroblastoma models. (**a**) Chemical structure of SRX3177. (**b**, **c**) Mantle cell lymphoma, Jeko; Hepatocellular carcinoma, Huh7 or NeuroblastomaCHLA-255 were treated with either SRX3177 or palbociclib at the IC_50_ or IC_10_ and assessed after 24 h by flow cytometry for FITC-annexin V and propidium iodide labeling. (**d**–**f**) NGS mice (n = 8 per group) harboring Huh7 or Jeko-1 subcutaneous xenografts were treated with SRX3177 (30 mg/kg for Huh7 or 40 mg/kg for Jeko-1) or vehicle control by oral gavage once daily dose for 1 week and assessed for tumor volume and total body weight. Additional groups (n = 8) were treated with equal weight per volume doses of JQ1, palbociclib, and BK120 combination therapy versus respective vehicles and assessed for mortality. Asterisks denote *p* ≤ 0.05 in comparison with vehicle control by unpaired Student’s *t* test.

The differences in structures of the three complexes arise in coordination of substituents. While the nitrogen atom in the piperazine ring of SRX3212P and SF2523P is unrestrained, the oxygen atom in the morpholine ring of SRX3212 is involved in a water mediated polar interaction with the carboxyl group of Asp144, which likely accounts for a sevenfold increase in potency of SRX3212 compared to SRX3212P. Although the hydrogen bond between the side chain amide of Gln85 and the dioxane moiety of the thienopyranone scaffold is conserved, restraining SRX3212, SRX3212P and SF2523P, the presence of the pyrid-3-ylmethylaminocarbonyl benzodioxane moiety in SRX3212 and SRX3212P altered the hydrophobic contacts with Trp81. The indole ring of Trp81 flips over ~ 180° in SRX3212 and SRX3212P compared to this ring’s position in SF2523P, enhancing its interaction with the benzodioxane moiety. In support, a large difference in chemical shift perturbations (CSPs) of the side chain NH^ε^ group of Trp81 was observed in ^1^H,^15^N heteronuclear single quantum coherence (HSQC) titration experiments (Fig. 1d). The short 3.5 Å distance between the pyridyl moiety of SRX3212 and the methyl group of Leu92 suggested an additional methyl-π contact that might explain the enhancement in potency of SRX3212.

#### Structural basis of selectivity for BRD4_BD1_

To establish the molecular basis for high selectivity, we compared binding of inhibitors to BRD4 BDs by NMR. Gradual addition of SRX3212 or SRX3212P to the ^15^N-labeled BRD4_BD2_ NMR samples led to CSPs in the slow to intermediate exchange regime on the NMR time scale, revealing tight binding (Fig. 1f, left, and data not shown). However, SF2523P induced CSPs in the fast exchange regime indicative of a weak interaction in the micromolar range (Fig. 1f, right). In support of IC_50_ values, the NMR titration data demonstrate that while adding the pyrid-3-ylmethylaminocarbonyl substituent causes a moderate increase in selectivity toward BRD4_BD1_, the piperazine substituent appears to be the major driving force of the selectivity. Despite many attempts to determine structures of inhibitors bound to BRD4_BD2_, we were unable to obtain workable datasets, and therefore compared the structures of the BRD4_BD1_:SRX3212 and BRD4_BD1_:SRX3212P complexes with that of the BRD4_BD2_:SF2558HA complex^13^. The structural overlay shows that His437 of BRD4_BD2_ donates a hydrogen bond to the oxygen atom of the morpholine group of SF2558HA (Fig. 1e and Suppl. Figure 3, red dashed line). Unlike the morpholine oxygen, the nitrogen atom in the piperazine group of SRX3212P cannot serve as a hydrogen bond acceptor, and the lack of this contact with His437 may play a role in reducing the binding selectivity of this compound to BRD4_BD2_.

#### Inhibition of BRD4_BD1_ is not sufficient to provide strong anti-cancer effects

Although many inhibitors of BRD4 have been developed, the importance of inhibition of both BDs to subdue pathological processes remains unclear, as the two bromodomains have high sequence similarity. We therefore examined whether inhibition of only one bromodomain of BRD4 could lead to sufficient anti-cancer effects. Because SRX3212P is a potent inhibitor of BRD4_BD1_ but not BRD4_BD2_ or PI3K, whereas SRX3212 is a potent inhibitor of both BDs and PI3K, we measured and compared cytotoxic activity of these inhibitors in three cyclinD1 dependent mantle cell lymphoma cell lines (Jeko-1, Mino and Z138), a colon carcinoma cell line (HCT116), a MYCN amplified p53 mutated neuroblastoma cell line (SKNBE2) and a PTEN mutated prostate cancer cell line (PC3) (Fig. 1h). The dose response curves generated from CellTiter-Glo luminescent cell viability assays in these cell lines revealed strong anti-cancer activity of SRX3212. The IC_50_ values of SRX3212 were in the nanomolar range for all mantle cell lymphoma cells tested and in the low micromolar range for colon carcinoma and neuroblastoma cells, however the anti-proliferative activity of SRX3212P was markedly lower. Neither inhibitor sensitized the prostate cancer cell line PC3. Immunoblotting of lysates from JeKo-1 cells stimulated with IgM and treated with increasing concentrations of SRX3212 showed a concomitant decrease in levels of AKT phosphorylation at Ser473, pointing to the decrease in PI3K activity (Fig. 1g). Inhibition of PI3K by SRX3212 was comparable to inhibition of this kinase by its well-known inhibitor BKM120. In contrast, treatment of JeKo-1 cells with SRX3212P even at high concentration of 5 µM did not decrease AKT phosphorylation. Collectively, these data suggest that inhibition of only one bromodomain BRD4_BD1_ in the absence of PI3K inhibition does not provide desirable anti-proliferative effects and requires inhibition of both BDs. These results also corroborate the idea that concurrent inhibition of multiple pathological pathways, such as BRD4 and PI3K, increases efficacy.

#### TP triple BRD4/PI3K/CDK4/6 inhibitor induces cell cycle arrest and apoptosis

In addition to PI3K, several other signaling pathways, including CDK4/6, have been shown to have a synthetic lethality link with BRD4. We have previously identified the TP inhibitor SRX3177 with a nanomolar potency against PI3Kα (IC_50_ = 79 nM), both bromodomains of BRD4 (BD1 and BD2) (IC_50_ = 33 nM and 89 nM, respectively), and CDK4/6 (IC_50_ = 2.5/3.3 nM)^22^ (Fig. 2a). Given the synergistic relationship of BRD4, PI3K and CDK4/6 as cell cycle regulators, we tested the ability of SRX3177, capable of targeting BRD4, PI3K and CDK4/6 simultaneously, to induce cell cycle arrest in a cyclin D1-dependent hematologic malignancy (mantle cell lymphoma, JeKo-1), PI3K-dependent solid tumor (hepatocellular carcinoma, Huh7), and MYC-dependent embryonal tumor (neuroblastoma, CHLA-255) (Fig. 2b). Palbociclib was used as control given its known inhibition of CDK4/6 and resultant G1 cell cycle growth arrest. Propidium iodide flow cytometry analysis revealed induction of G1 cell cycle arrest at a low dosage of SRX3177 (near the IC_10_), with the most robust effects observed in hepatocellular carcinoma and mantle cell lymphoma cells. However, higher doses of SRX3177 (near the IC_50_) induced G2 phase growth arrest in these cell lines (Fig. 2b). We next assessed the level of apoptosis by FITC-annexin V incorporation in each of these cell lines. In response to treatment with SRX3177 at the IC_50_, both early and late apoptosis were markedly (~ 5–tenfold) induced in JeKo-1 mantle cell lymphoma cells and early apoptosis was induced ~ threefold in Huh7 hepatocellular carcinoma cells (Fig. 2c). Together, these data demonstrate that SRX3177 induces both G1 and G2 cell cycle arrest and apoptosis in a variety of tumor models.

#### TP triple inhibitor prevents growth of tumor xenografts

Because SRX3177 shows robust anti-cancer activity in vitro, we evaluated its efficacy using in vivo xenograft models. NGS mice harboring subcutaneous xenografts of Huh7 or JeKo-1 cells were treated with SRX3177 by oral gavage. After one week of treatment at either 30 mg/kg (Huh7) or 40 mg/kg (JeKo-1), there was significant abrogation of tumor growth in SRX3177 treated animals compared to vehicle control treated animals (Fig. 2d). Animal weight remained the same in both groups, and no adverse effects were noted in the SRX3177 treated animals to suggest any other toxicity (Fig. 2e). In contrast, mice treated with a combination of BKM120 (PI3K inhibitor), palbociclib (CDK4/6 inhibitor), and JQ1 (BET inhibitor) showed effects of significant toxicity. Mortality for this group of animals was 20–40%, whereas no treatment-related death was observed in the SRX3177 treated group (Fig. 2f). Collectively, these data demonstrate that the multi-target TP inhibitor SRX3177 has antitumor efficacy in in vivo xenograft models and is less toxic than the combination of agents that inhibit individual targets.

#### Concluding remarks

In this study, we highlight the structural and molecular basis underlying enhanced potency and selectivity of thienopyranone-based BRD4 inhibitors. We have developed and characterized one of the most potent BRD4 inhibitor to date, SRX3212, with an IC_50_ of 3.7 nM for BRD4_BD1_. Comparative analysis presented here broadens our understanding of the role of individual BRD4 bromodomains in cell proliferation and oncogenic processes*.* The data suggest that inhibition of only BRD4_BD1_ is not efficient enough to provide strong anti-proliferative effects in the cancer cell lines tested, including mantle cell lymphoma, colon cancer and neuroblastoma cell lines. Furthermore, decreased BRD4_BD2_ selectivity is also accompanied by decreased PI3K selectivity. In contrast, simultaneous targeting of multiple pathological pathways, implicating both BDs of BRD4 and additional cancer drivers such as PI3K and/or CDK4/6 notably increases efficacy.

We have previously shown that SF2523 blocks tumor immunosuppression, restores CD8^+^ T-cell activity and promotes adaptive immune responses in cancer^16^. Furthermore, we have demonstrated that SF2523 decreases human immunodeficiency type-1 (HIV) replication in macrophages via degradation of intracellular HIV through autophagy^23^. Because BRD4 has emerged as a protein target for the SARS-CoV-2 virus envelope protein E^6^, the TP-based inhibitors, and particularly the most potent compound SRX3212, could provide critical tools in determining the role of BRD4 in viral infections, including SARS-CoV types. Together, these findings support the advancement of the novel TP-based dual and triple inhibitors reported for further investigation in personalized therapeutics for immuno-oncology and viral diseases.

## Experimental procedures

All methods were carried out in accordance with relevant guidelines and regulations.

### Protein expression and purification

The BRD4 pGEX6p-1 bromodomain 1 (43–180) or pGEX4T-1 bromodomain 2 (342–460) constructs were transformed into Escherichia coli BL21 RIL cells. The cells were cultured at 37 °C using Luria Broth or M19 minimal media supplemented with 15N-NH_4_Cl, induced at an OD_600_ ~ 0.6 with a final concentration of 0.5 mM IPTG and cultured overnight at 18 °C. Cell cultures were harvested by centrifugation at 5,000 rpm and resuspended in 50 mM HEPES pH 7.5, 150 mM NaCl and 1 mM diothiothreitol (DTT). Resuspended cells were lysed by freeze–thaw followed by sonication. Uniformly ^15^N-labeled and unlabeled proteins were purified on glutathione Sepharose 4B beads and the GST tag was cleaved with PreScission or thrombin protease. The cleaved protein was concentrated using a 3 kDa CO concentrator and further purified by HPLC using a HiPrep Sephacryl S-100 h column (GE) in 10 mM HEPES pH 7.5, 100 mM NaCl, 1 mM TCEP. Protein fractions were checked by SDS-PAGE and concentrated to ~ 10–20 mg/ml.

### Measurements of IC_50_

IC_50_ measurements for inhibition of His-tagged BRD4_BD1_ and BRD4_BD2_ by SF2523P, SRX3212 or SRX3212P (synthesis of the inhibitors will be described elsewhere) were performed by Reaction Biology using an AlphaScreen assay with tetra-acetylated histone H4 peptide (1–21) (H4K5ac/8ac/12ac/16ac-Biotin) as a ligand. PI3K activity screening and IC_50_ measurements were performed by Life Technologies (Thermo Fisher Scientific) using ADAPTA, a fluorescence-based in vitro assay.

### Nuclear magnetic resonance (NMR)

NMR experiments were carried out at 298 K on a Varian INOVA 600 MHz spectrometer equipped with a cryogenic probe. The ^1^H,^15^N heteronuclear single quantum coherence (HSQC) spectra of 0.2 mM uniformly ^15^N-labeled BRD4_BD1_ and BRD4_BD2_ were collected in the presence of increasing concentrations of either SF2523P, SRX3212 or SRX3212P in PBS buffer, pH 6.8, 8% D_2_O. NMR data were processed with NMRPipe. NMR assignments are taken from^24^.

### X-ray crystallography

BRD4_BD1_ (43–180) was incubated with 2–3 molar equivalence of SF2523P, SRX3212 or SRX3212P at RT for 30 min prior dialysis against 10 mM HEPES pH 7.5, 100 mM NaCl, 1 mM TCEP. The dialyzed protein-inhibitor mixtures were concentrated to 6–9 mg/ml. Crystals of BRD4_BD1_ in complex with SF2523P and SRX3212P were grown by the sitting-drop vapor diffusion method in a 1.6 µl drop using 1:1 ratio of protein/inhibitor : reservoir solution at 18 °C and seeding post-equilibrium. Crystals of BRD4_BD1_ in complex with SF2523P were formed in a drop containing reservoir solution (2.5 M Ammonium Sulfate and 0.1 M Tris pH 8.5). Crystals of BRD4_BD1_ in complex with SRX3212P were formed in a drop containing reservoir solution (25% PEG 3,350, 0.2 M NH_4_Cl, 0.1 M Tris pH 8.5). Crystals of BRD4_BD1_ in complex with SRX3212 were formed in a similar drop composition containing reservoir solution (40% PEG 3,350, 0.2 M Potassium thiocyanate pH 7.5, and 4% 1,1,1,3,3,3-Hexafluoro-2-propanol) at 4 °C. X-ray diffraction datasets for the complexes were collected at 100 K on a Rigaku Micromax 007 high-frequency microfocus X-ray generator with a Pilatus 200 K 2D area detector (University of Colorado Anschutz X-ray core facility). The data were indexed and scaled using HKL2000/3000^25^. Phase solutions were determined by molecular replacement in Phaser^26^ using BRD4_BD1_ (PDB IDs: 3mxf or 5U28) as a search model with waters and ligands removed. Refinement was performed in PHENIX Refine^27^ and manually by Coot^27^.

### Cell viability assay

All cell lines were obtained from ATCC and tested for mycoplasma and mouse pathogens and checked for authenticity against the International Cell Line Authentication Committee (ICLAC; https://iclac.org/databases/cross-contaminations/) list. Cell viability measurements were performed using CellTiter-Glo luminescent cell viability assay (Promega). JeKo-1, Mino and Z138 cells were plated in 96 well plates at a density of 1 × 10^4^ in 100 µl media whereas HCT116, SKNBE-2 and PC3 cells were seeded at a density of 2 × 10^3^ in 96 well plate followed by incubating with different concentration of SRX3212 and SRX3212P for 48 h. Cell Titer Glow assay reagent (100 µl) was added to each well and incubated for 10 min at room temperature. Luminescence signal was measured in a Varioscan multimode reader.

### Western blot analysis

JeKo cells (1 × 10^6^) were serum starved for 6 h and incubated with or without SRX3212 and SRX3212P for 1 h. Cells were resuspended with 1 ml of RPMI-1640 (serum free) and stimulated with 10 µg/ml of goat F(ab) 2 anti-human IgM antibody (Southern Biotech Birmingham, AL, USA) at 37 °C for 10 min. Cells were then harvested and lysed with RIPA buffer followed by Western blot analysis for pAKT expression.

### Cell cycle and apoptosis assays

Huh7, CHLA-255, or Jeko-1 cells were seeded at 1.5 × 10^6^ cells in 60-mm tissue culture dishes and allowed to adhere overnight. Cells were treated for 24 h with SRX3177 or palbociclib at concentrations comparable to the IC_50_ or IC_10_ for each compound and cell line. Cells were released with Accutase (Innovative Cell Technologies, San Diego, CA), washed with PBS and divided for either staining with the FITC Annexin V Apoptosis Detection Kit (BD Biosciences, San Jose, CA) or overnight fixation with ethanol followed by staining with propidium iodide (Roche, Basel, Switzerland) according to each manufacturers’ instructions. Cell staining was analyzed with the FACSCalibur flow cytometer (BD Biosciences) and FlowJo (FlowJo LLC, Ashland, Oregon) and ModFit LT (Verity Software House, Topsham, ME) software packages.

### Animal studies

All procedures involving animals were approved by the UCSD Animal Care Committee, which serves to ensure that all federal guidelines concerning animal experimentation are met. Six to eight week old NGS mice were implanted with 10^7^ Huh7 or Jeko-1 cells in the right flank. When tumors reached ∼100 mm^3^ after tumor implantation, animals were divided into four groups (n = 8 per group). Group 1 was treated with Hot Rod vehicle (Catalent, Somerset, NJ), group 2 was treated with SRX3177, group 3 was treated with NMP/PEG300 (10/90 v/v) (BMK120 vehicle) plus lactated Ringer’s (pH 4.0) (palbociclib vehicle) plus 0.5% methylcellulose and 0.2% Tween 80 (JQ1 vehicle), and group 4 was treated with BKM120 plus palbociclib plus JQ1. Mice were treated for 1 week by oral gavage, 5 times per week.

### Statistical analysis

All statistical analysis was performed using Microsoft Excel. The Student’s *t* test was used to evaluate differences observed between experimental groups. Statistical significance was accepted at a p-value less than 0.05.

## Supplementary information

Supplementary Information.

## Data Availability

The atomic coordinates and structure factors have been deposited in the Protein Data Bank under accession codes 6X7B, 6X7C, and 6X7D. All other relevant data supporting the key findings of this study are available within the article and its Supplementary Information files or from the corresponding authors upon reasonable request.

## References

[1] Filippakopoulos P (2010). Selective inhibition of BET bromodomains. Nature.

[2] Filippakopoulos P, Knapp S (2014). Targeting bromodomains: epigenetic readers of lysine acetylation. Nat. Rev. Drug Discov..

[3] Lambert JP (2019). Interactome rewiring following pharmacological targeting of BET bromodomains. Mol. Cell.

[4] Belkina AC, Nikolajczyk BS, Denis GV (2013). BET protein function is required for inflammation: Brd2 genetic disruption and BET inhibitor JQ1 impair mouse macrophage inflammatory responses. J. Immunol..

[5] Nicodeme E (2010). Suppression of inflammation by a synthetic histone mimic. Nature.

[6] Gordon DE (2020). A SARS-CoV-2-human protein–protein interaction map reveals drug targets and potential drug-repurposing. Nature.

[7] Filippakopoulos P (2012). Histone recognition and large-scale structural analysis of the human bromodomain family. Cell.

[8] Shi J (2014). Disrupting the interaction of BRD4 with diacetylated twist suppresses tumorigenesis in basal-like breast cancer. Cancer Cell.

[9] Zhang P (2017). Intrinsic BET inhibitor resistance in SPOP-mutated prostate cancer is mediated by BET protein stabilization and AKT-mTORC1 activation. Nat. Med..

[10] Dai X (2017). Prostate cancer-associated SPOP mutations confer resistance to BET inhibitors through stabilization of BRD4. Nat. Med..

[11] Rathert P (2015). Transcriptional plasticity promotes primary and acquired resistance to BET inhibition. Nature.

[12] Fong CY (2015). BET inhibitor resistance emerges from leukaemia stem cells. Nature.

[13] Andrews FH (2017). Dual-activity PI3K-BRD4 inhibitor for the orthogonal inhibition of MYC to block tumor growth and metastasis. Proc. Natl. Acad. Sci. U. S. A.

[14] Stratikopoulos EE (2015). Kinase and BET inhibitors together clamp inhibition of PI3K signaling and overcome resistance to therapy. Cancer Cell.

[15] Ciceri P (2014). Dual kinase-bromodomain inhibitors for rationally designed polypharmacology. Nat. Chem. Biol..

[16] Joshi S (2019). SF2523: Dual PI3K/BRD4 inhibitor blocks tumor immunosuppression and promotes adaptive immune responses in cancer. Mol. Cancer Ther..

[17] Carlino L, Rastelli G (2016). Dual kinase-bromodomain inhibitors in anticancer drug discovery: a structural and pharmacological perspective. J. Med. Chem..

[18] Wang NY (2020). Design, synthesis, and biological evaluation of 4,5-dihydro-[1,2,4]triazolo[4,3-f]pteridine derivatives as novel dual-PLK1/BRD4 inhibitors. Eur. J. Med. Chem..

[19] He S (2020). Potent dual BET/HDAC inhibitors for efficient treatment of pancreatic cancer. Angew. Chem. Int. Ed. Engl..

[20] Yan Y (2019). The novel BET-CBP/p300 dual inhibitor NEO2734 is active in SPOP mutant and wild-type prostate cancer. EMBO Mol. Med..

[21] Watts E (2019). Designing dual inhibitors of anaplastic lymphoma kinase (ALK) and bromodomain-4 (BRD4) by tuning kinase selectivity. J. Med. Chem..

[22] Burgoyne AM (2020). A triple action CDK4/6-PI3K-BET inhibitor with augmented cancer cell cytotoxicity. Cell Discov..

[23] Campbell GR (2018). Induction of autophagy by PI3K/MTOR and PI3K/MTOR/BRD4 inhibitors suppresses HIV-1 replication. J. Biol. Chem..

[24] Abner E (2018). A new quinoline BRD4 inhibitor targets a distinct latent HIV-1 reservoir for reactivation from other “shock” drugs. J. Virol..

[25] Minor W, Cymborowski M, Otwinowski Z, Chruszcz M (2006). HKL-3000: the integration of data reduction and structure solution–from diffraction images to an initial model in minutes. Acta Crystallogr. D Biol. Crystallogr..

[26] McCoy AJ (2007). Phaser crystallographic software. J. Appl. Crystallogr..

[27] Adams PD (2010). PHENIX: a comprehensive Python-based system for macromolecular structure solution. Acta Crystallogr. D Biol. Crystallogr..

