# Supplementary material for: Design of thienopyranone-based BET inhibitors that bind multiple synthetic lethality targets
Source: Sci Rep. 2020 Jul 21;10:12027. doi: 10.1038/s41598-020-68964-6 (PMC7374098; doi:10.1038/s41598-020-68964-6)
Supplement: Supplementary file 1 — Supplementary Information. [file 41598_2020_68964_MOESM1_ESM.docx]

**Supplementary Information**

**Design of thienopyranone-based BET inhibitors that bind multiple synthetic lethality targets**

Kendra R. Vann^1,4^, Dhananjaya Pal^2,4^, Guillermo A. Morales^3^, Adam M. Burgoyne^2^, Donald L. Durden^2,3*^ and Tatiana G. Kutateladze^1*^

^1^Department of Pharmacology, University of Colorado School of Medicine, Aurora, CO, USA

^2^Division of Pediatric Hematology and Oncology, Department of Pediatrics, Moores Cancer Center, University of California San Diego, La Jolla, CA, USA

^3^SignalRx Pharmaceuticals, San Diego, CA, USA

^4^Equal contribution

^*^Correspondence: Tatiana G. Kutateladze, [tatiana.kutateladze@cuanschutz.edu](mailto:tatiana.kutateladze@cuanschutz.edu) or Donald L. Durden, ddurden@ucsd.eduen@ucsd.edu

**
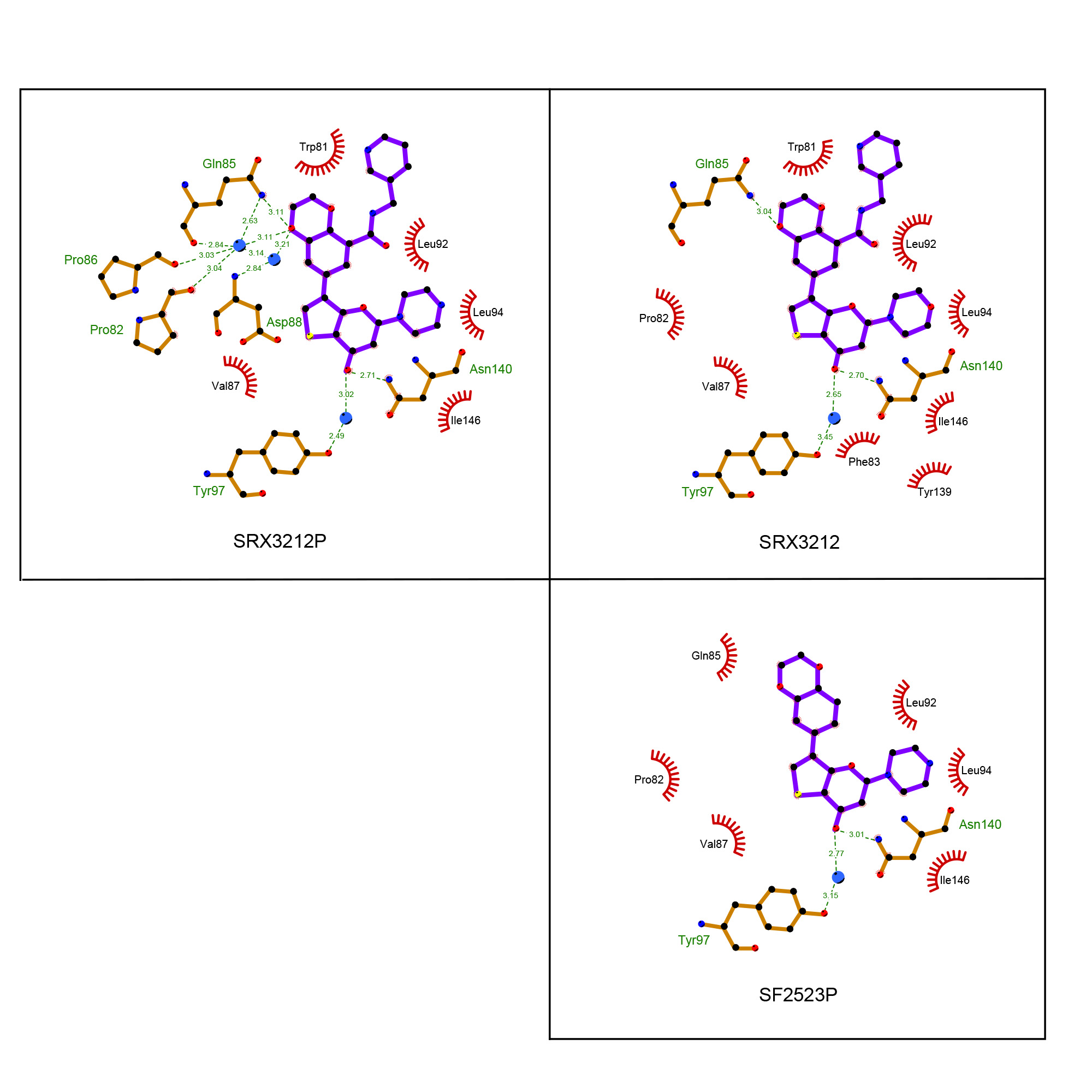
Supplementary Figure 1.** Two-dimensional LigPlot schematics of BRD4_BD1_ with indicated bound inhibitors. Hydrophobic interactions are represented by red arcs. Electrostatic interactions are indicated by green dashed lines. Water molecules are indicated by blue spheres. Related to Figure 1.

**
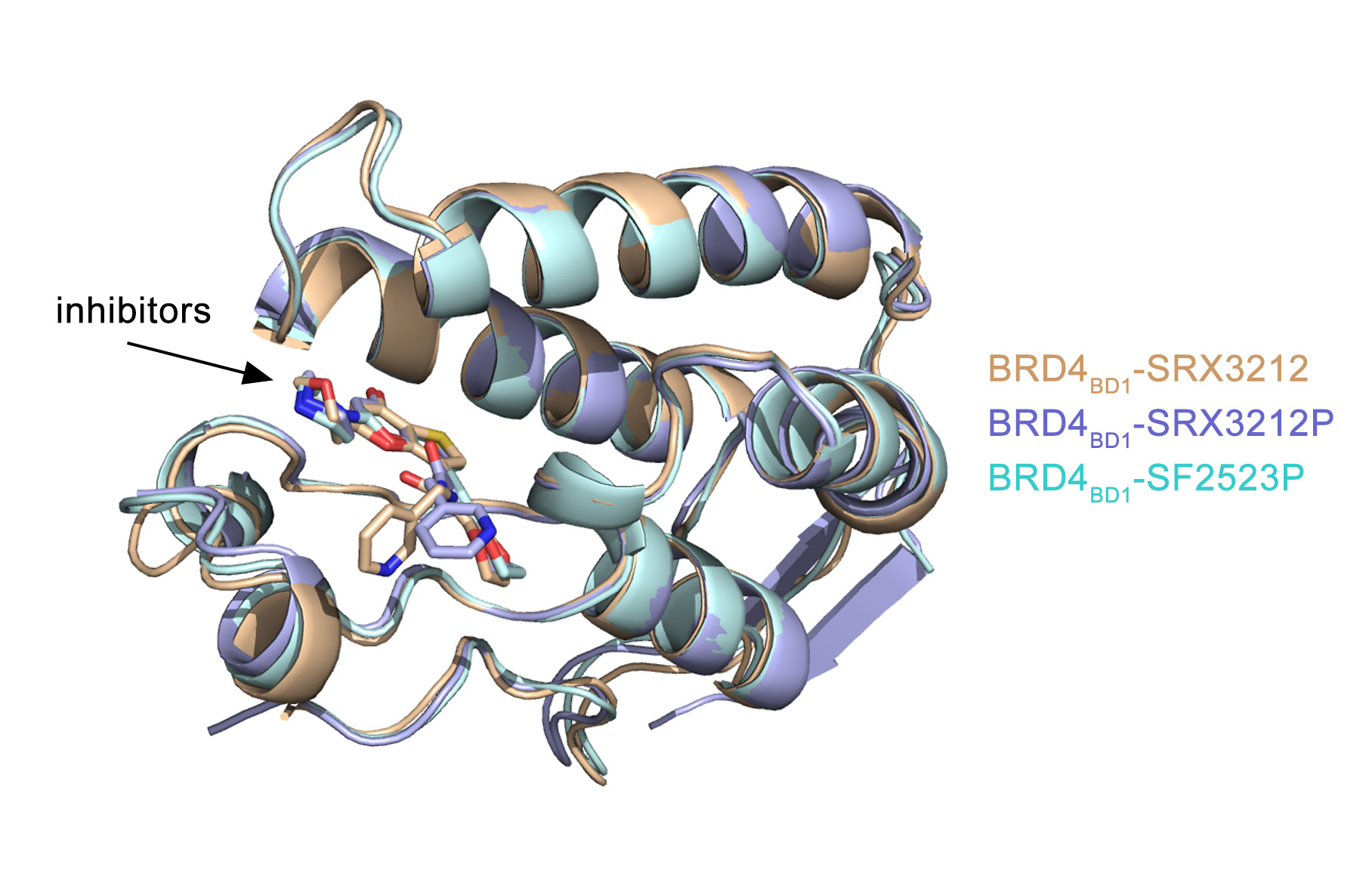
**

**Supplementary Figure 2.** Overlay of crystal structures of BRD4_BD1_ in complex with SRX3212 (tan), SRX3212P (purple) and SF2523P (cyan). Related to Figure 1.

**
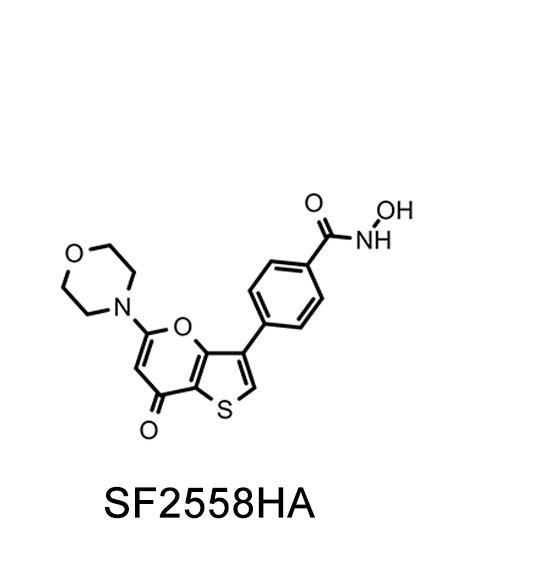
**

**
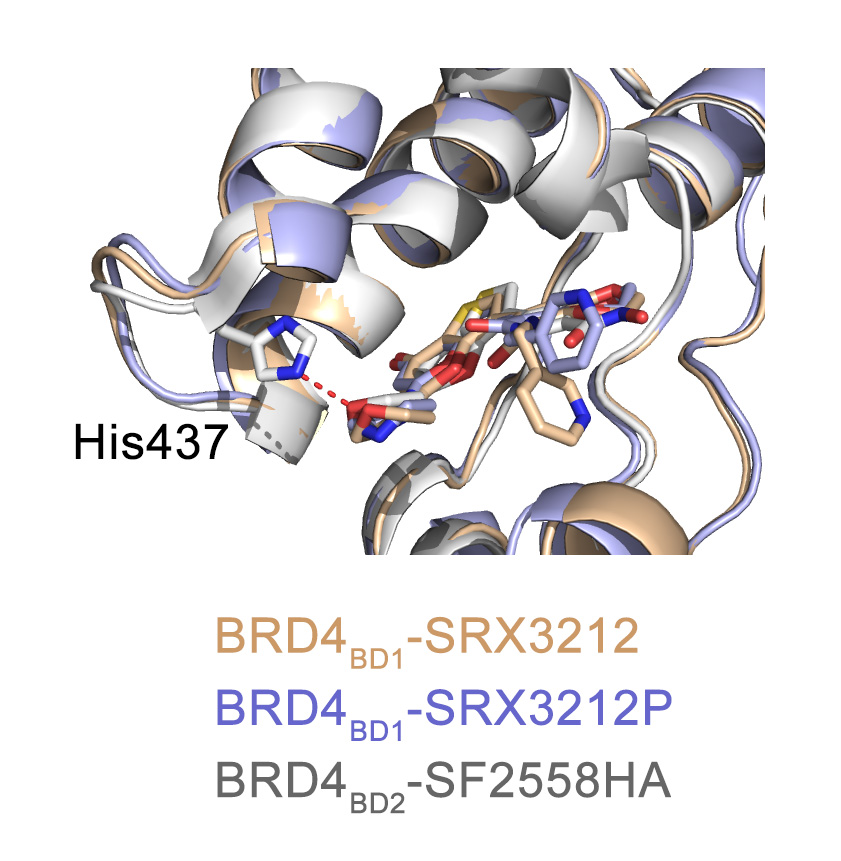
**

**Supplementary Figure 3.** Overlay of crystal structures of BRD4_BD1_ in complex with SRX3212 (tan) and SRX3212P (purple) and of BRD4_BD2_ in complex with SF255HA (gray) (PDB ID: 5U2C). Chemical structure of SF2558HA is shown above. Related to Figure 1.

| **Table 1. Data Collection and Refinement Statistics** | | | |
| --- | --- | --- | --- |
|  | **BD1 in complex with**  **SF2523P** | **BD1 in complex with**  **SRX3212** | **BD1 in complex with SRX3212P** |
| **Data Collection** |  |  |  |
| Space group | P 2_1_ | P 1 | P 2_1_ |
| Wavelength (Å) | 1.54 | 1.54 | 1.54 |
| Resolution (Å) | 50.00-2.5 (2.59-2.5)* | 50.00- 2.70 (2.80-2.70)* | 50.00- 1.95 (2.02-1.95)* |
| Unit-cell dimensions  *a, b, c* (Å)  α,β,γ (°) | 56.15, 39.83, 62.04  90.0, 106.5, 90.0 | 30.55, 34.65, 36.03 62.9, 83.6, 85.5 | 56.24, 39.45, 62.12  90.0, 108.0, 90.0 |
| No. of measured reflections | 29239 | 7734 | 60665 |
| No. of unique reflections | 8982 | 3407 | 19048 |
| Redundancy | 3.3 (3.1) | 2.3(2.3) | 3.2 (2.7) |
| I/σ | 15.9 (4.0) | 10.9 (12.3) | 16.5 (5.5) |
| Completeness (%) | 95.5 (97.4) | 94.9 (94.4) | 99.1 (93.3) |
| R_sym_^#^ (%) | 9.9 (34.9) | 6.2 (8.2) | 6.0 (19.1) |
| R_pim_^#^ (%) | 6.0 (21.4) | 4.9 (6.2) | 3.9 (13.4) |
| No. of molecules in ASU | 2 | 1 | 2 |
| Solvent content (%) | 41.2 | 46.3 | 46.4 |
| **Refinement** |  |  |  |
| R_work_/R_free_ (%) | 19.53/26.84 | 20.02/25.69 | 20.01/22.11 |
| No. of atoms | 2294 | 1176 | 2494 |
| Protein | 2126 | 1063 | 2221 |
| Ligand/ion | 52 | 36 | 72 |
| Water | 116 | 77 | 201 |
| B-factors (Å^2^) | 38.48 | 23.70 | 22.70 |
| Protein | 38.71 | 23.71 | 22.65 |
| Ligand/ion | 35.28 | 25.28 | 22.26 |
| Water | 35.72 | 22.82 | 23.39 |
| R.M.S.D |  |  |  |
| Bond lengths (Å) | 0.009 | 0.004 | 0.004 |
| Bond angles (°) | 1.02 | 0.69 | 0.94 |
| Ramachandran favored (%) | 96.4 | 98.4 | 100.0 |
| Ramachandran allowed (%) | 3.6 | 1.6 | 0.0 |
| Ramachandran outliers | 0 | 0 | 0 |
| Clashscore | 14.43 | 6.04 | 2.88 |

*Values in parentheses are for the highest resolution shell (Å).

^#^R_sym_ =∑|*I*_obs_-*I*_avg_|/I_avg,_ where I_obs_ is intensity of any given reflection and I_avg_ is the weighted mean I.
